# Dr. Galezowski’s Binocular Strabometer

**Published:** 1869-08

**Authors:** 


### Dr. Galezowski's Binocular Strabometer.

M. Beclard presented to the Academy of Medicine of Paris, on the 23d ult., a very ingenious and simple little instrument invented by Dr. Galezowski,—a binocular strabometre. Every surgeon knows how important it is, in the operation of strabismus, to measure with exactitude the degree of deviation as well as the precise result obtained by tenotomy. With the strabometre of Dr. Laurence, which has to be moved from one eye to the other in order to compare the degree of deviation, this is often very difficult. The binocular strabometre of Galezowski does not present the same inconvenience; the two needles, which slide in the sulcus of a screw, are easily placed opposite to the centre of each cornea; and by the divisions which are marked upon the horizontal bar, we immediately note the deviation. The instrument is composed of a horizontal branch, upon which slide two needles destined to indicate the degrees, and which place themselves opposite to the centre of each corresponding cornea. This transverse bar is held on a level with the eye-lids, the handle of the instrument upwards, and the fork of the bar against the root of the nose. On turning the little buttons attached to the extremities of the bar, the needles move from right to left and left to right, until each one is found to be opposite the centre of the pupil. The graduation of the transverse bar gives us the degree of deviation with the greatest precision. The handle of the instrument might be omitted, which would leave a mere horizontal bar, thus reducing it to the greatest simplicity.—*New Orleans Journal of Medicine.*

